# Exploring the impact of stimulus–stimulus and stimulus–response conflicts on computer mouse trajectories: continuous flow of information from stimulus encoding to response preparation to motor action

**DOI:** 10.1007/s00426-023-01840-w

**Published:** 2023-06-01

**Authors:** Hayley Tseng, Markus F. Damian

**Affiliations:** School of Psychological Science, University of Bristol, 12a Priory Road, Bristol, BS8 1TU UK

## Abstract

In recent years, mouse tracking (designing experiments in which participants provide responses via dynamic computer mouse movements) has enjoyed increasing experience in experimental psychology. Mouse-tracking studies typically involve some form of stimulus–response (S–R) conflict, and S–R effects emerge in movement trajectories (as well as in latencies). By contrast, it is currently unclear how stimulus–stimulus (S–S) compatibility affects movements. Here, we used a spatial arrow task which allowed us to generate S–R and S–S effects within the same experiment. Experiment 1 clarified in a key press experiment that this manipulation generates clear S–S and S–R effects in latencies. More critically, Experiment 2 demonstrated that both types of conflict impact mouse trajectories with incompatibility emerging as increased ‘curvature’ of responses when compared to congruent responses. We argue that these results are best explained via the assumption of ‘continuous flow’ of information, from stimulus encoding to response preparation and finally into motor action. By contrast, the S–S effect on trajectories contradicts the notion that processing is ‘thresholded’ between stimulus encoding and response preparation.

## Introduction

Being able to operate efficiently in daily life requires the ability to orient our attention and behaviour towards goal-relevant information under conditions of conflict (‘cognitive control’). The mechanisms behind cognitive control have been extensively explored with tasks that involve some form of conflict. For instance, in the Simon task, a stimulus is classified into one of two responses (e.g., by colour: blue → left; red → right) and the stimulus is presented to the left or right of the screen. The irrelevant stimulus position is compatible or incompatible with the response side, and reaction times are typically slowed on incompatible compared to compatible trials (see Hommel, 2011, for review). Cognitive control is required to resolve the incompatibility on incongruent trials, and subtracting reaction times in the congruent trials from reaction times in the incongruent trials provides a measure of the effectiveness of an individual’s ability to exert cognitive control.

In conflict tasks, relevant and irrelevant stimulus dimensions and the response can engage in various forms of congruency. The ‘dimensional overlap’ (DO) model by Kornblum (1994) systematically organises various experimental tasks into a taxonomy of ‘ensembles’ depending on their dimensional overlap, with the Simon task a member of pure stimulus–response (S–R) (or ‘Ensemble 3’) tasks. However, congruency effects can also arise from instances in which the response dimension is not involved, but instead relevant and irrelevant stimulus dimensions overlap. An instance of a stimulus-stimulus (S–S, or ‘Ensemble 4’ type) task is the classic colour-word Stroop task with verbal responses, in which the conflict in the ‘incongruent’ condition (e.g., naming the colour of the word BLUE printed in red) arises from two overlapping stimulus dimensions. It is worth highlighting that even in tasks characterised as involving ‘stimulus-stimulus’ conflicts here and elsewhere, it is still the case that responses might compete against each other. Even in the Stroop task, co-activated words compete with each other in the mental lexicon (e.g., Roelofs, 2003). Hence, any task which involves some sort of decision can be characterised as having a ‘response dimension’. Here we use the term stimulus–stimulus conflict in the narrower sense that relevant and irrelevant dimensions overlap regarding their stimulus but not their response characteristics (Kornblum, 1994). This issue will be unfolded in greater detail in the General Discussion.

Kornblum’s classification of stimulus/response compatibility constellations have had considerable impact because it relates experimental tasks to theoretical notions of perceptual and cognitive processing. His DO model (Kornblum, 1994) joins a tradition of models prevalent at the time (e.g., Sternberg, 1969) according to which information processing consists of sequence of stages, with strict thresholding in between them. Hence, information processing at a particular stage is not initiated until processing at the previous stage has been completed to a point where a critical threshold has been reached. For instance, in Kornblum et al.’s (1999) computational implementation of the DO model, processing is divided between an input layer (concerned with stimulus encoding) and an output layer (in charge of response preparation), with a thresholding mechanism in between the two stages which gives the model serial processing characteristics. As a theoretical alternative to such strictly serial models, information may continuously ‘leak’ (or cascade) from one stage to the next, a notion which is embedded in numerous frameworks arising from the ‘parallel distributed processing’ framework (e.g., McClelland, 1979). In recent years, these continuous models have been favoured over thresholding models.

### The characteristics of S–S and S–R effects

Over the last few decades various empirical approaches have been developed to explore whether S–S and S–R conflict are resolved at different stages. According to the DO model, the former is processed in the stimulus encoding stage while the latter is processed in the response preparation stage. If we are to assume that this is the case, then we should expect to observe distinct characteristics of each conflict type. Indeed, there has been evidence to indicate that the S–S and S–R effects are associated with contrasting temporal dynamics. For example, Kornblum (1994) found that the S–R effect was larger when the relevant and irrelevant stimulus were presented simultaneously than when the irrelevant stimulus was displayed first. The reverse result was observed for S–S effects and hence, this difference indicates that S–S and S–S processing undergo contrasting time courses.

However, one could argue that demonstrating contrasting temporal dynamics between S–S and S–R conflicts is not sufficient evidence to suggest that these conflicts are resolved at different stages. Other strategies to distinguish between S–S and S–R conflict include factorial crossing the various types of compatibility manipulations and correlating the conflict scores with one another. The main assumption of the first strategy is that findings of additivity would indicate the two conflict types are resolved at different stages while interactivity implies that they are addressed within the same stage. Overall, the results relating to this strategy have been mixed. Some studies indicate strict additivity of both types of conflicts (e.g., Hommel, 1997, Exp. 1) whereas others exhibit underadditivity (e.g., Hommel, 1997, Exp.2). This inconsistency may be partially or wholly attributed to whether or not the ‘base’ S–S and S–R effects are substantial enough (Hommel, 1997; Rey-Mermet, 2020; Sanders, 1980). Another strategy of comparing S–S and S–R conflict is to correlate the effects produced from these conflicts with each other. Here the logic is that a substantial correlation would point to a shared locus of the effect, whereas separate loci might predict independence. To our knowledge, only two studies have used this strategy with ‘pure’ S–S and S–R tasks (Li et al, 2014; Paap et al, 2019). Li et al (2014) observed a null correlation on latencies but Paap et al (2019) reported a significant correlation on 'efficiency scores’, i.e., a composite measure of speed and accuracy.

In summary, stimulus and response dimensions can be set up to conflict with one another in experimental tasks in a variety of ways. However, the exact relation between S–S and S–R effects regarding their processing characteristics remains elusive and difficult to pinpoint. This is the case even though current theoretical models tend to favour cascaded/continuous information flow over serial, staged processing.

### The relationship between cognition and action

Similar issues arise when considering the relationship between cognition and action. A popular (and often tacit) assumption in cognitive psychology is that information flow between the final stage of cognitive processing, and of the motor stage, is thresholded: action is only released when cognitive processing has been completed. In fact, according to Calderon et al. (2018), most models, including continuous ones, follows this assumption. For instance, this notion is central to the family of ‘diffusion decision’ models (e.g., Ratcliff et al., 2016) according to which evidence for response alternatives accrues until it reaches a specific decision threshold for one of the responses. By contrast, the ‘Unfolding Action Model’ proposed by Calderon et al (2018) suggests that information from the cognitive stage can continue to leak into action after a movement is initiated.

In strictly behavioural work, the keypress method has been used alongside conflict tasks to assess cognitive control, with this ballistic response representing the end of a chain of processing stages involved in decision-making. With key presses as responses, arguably a response can only be executed after a decision has been completed. However, as argued by Wispinski et al. (2020), although it is acknowledged that data derived from such activities constitute an important source of information, a ballistic response “…does not reflect the vast majority of evolutionarily old and ecologically valid decisions for which the primate brain is organized […] competition occurs before and continues after movement initiation” (p. 40). Therefore, in our paper we will focus on studies which use ‘dynamic’ response methods to measure cognitive control, such as ‘mouse tracking’ or ‘reach tracking’ (see Erb et al., 2021; Schoemann et al., 2020; Wirth et al., 2020, for recent overviews). Dynamic response methods usually involve participants completing a conflict task where responses are made by either moving the computer mouse cursor from the bottom of the screen to one of two response areas located in the upper left and right corners of the screen (in mouse tracking studies; e.g., Ye & Damian, 2023), or participants lifting their finger from a starting position typically below a screen, and touching one of two response locations on a projector screen (in reach tracking studies, e.g., Erb & Marcovitch, 2018). Mouse trajectories or hand movements are recorded, and different types of response measures are generated. These include initiation times, movement duration and reaction times. Critically, mouse or hand movement path curvatures can be captured in ‘dynamic’ response methods. Curvatures can be taken to reflect the online influence of conflict, with straight trajectories indicating no or little conflict, whereas deflections away from the correct response suggesting the presence of conflict. Therefore, a fundamental advantage of using this method over the keypress method is that researchers can capture the interplay between cognition and action and in turn, are able to explore the dynamics of decision-making (Freeman et al., 2011; Schoemann et al., 2020).

On a very broad level, results from ‘dynamic’ response methods suggest considerable ‘leakage’ from cognition into action. Strictly serial processing would predict that motor action is only released once a decision has been fully completed. If so, the most likely outcome would be that motor activity in dynamic tasks is temporally variable in response to cognitive processing (i.e., prolonged decision making, perhaps as a result of a stimulus–response conflict, should slow down both response initiation and completion) but the shape of the response should be similar or identical. To the contrary, response curvatures are influenced by cognitive manipulations in a wide variety of contexts, which suggests that decision making affects response execution. This general observation is in line with contemporary cognitive theories in which mind, body and environment dynamically interact (e.g., Spivey & Dale, 2004).

One fundamental aspect of ‘dynamic’ response methods which so far has been unresolved will be explored in the work reported here. Virtually all studies have involved some form of stimulus–response conflict which in the case of incongruency, evokes a powerful tendency to provide the incorrect response. For instance, the Simon task involves an overlap of the irrelevant stimulus with the response dimension (S–R conflict). The Simon effect appears in response latencies of key press experiments (e.g., Simon & Rudell, 1967) but it also emerges in the curvatures of dynamic responses generated with the computer mouse (e.g., Scherbaum & Dshemuchadse, 2020; Scherbaum et al., 2010) which could plausibly reflect the simultaneous activation of competing responses during movement execution. Hence, cognition ‘leaks’ into motor action. By contrast, we are not aware of studies which explored dynamic responses in conjunction with ‘pure’ stimulus–stimulus manipulations. Does a stimulus–stimulus conflict affect response trajectories in a similar way to a stimulus–response conflict?

Empirically, this question has not been directly investigated. In a recent overview of dynamic methods which mainly centred on the Flanker task, Erb et al. (2021) wrote: “An important question for future research to address concerns the extent to which initiation times and reach curvatures are impacted by stimulus-level and response-level conflict […] …none of the hand-tracking studies reviewed above directly evaluated the relative contributions of stimulus- and response-level conflict” (p. 743). The authors suggested that some effects on curvature might not be exclusively due to response congruency. For instance, Erb et al. (2016) reported a ‘reach tracking’ Stroop task in which participants classified three colours (red, blue or green) of words by reaching towards three corresponding response locations (bottom left, top centre, and bottom right). The locations for the distractors were either semantically cued or non-cued by the target. For example, if the trial involved the word RED written in blue, the semantically cued distractor would be red and the non-cued distractor would be green. Words could either be congruent with their colour (RED printed in red) or incongruent (RED printed in blue), and trials were analysed dependent on colour-word congruency, as well as on the congruency on the previous trial N-1. The authors also calculated distractor attraction scores representing the degree in which trajectories were more curved towards the semantically cued distractors than the non-cued ones over time. When analysing the distractor attraction scores for incongruent trials where the previous trial was congruent, the authors observed more ‘attraction’ towards semantically cued distractors than non-cued distractors and hence, demonstrating response-level conflict. Furthermore, when the previous trial was incongruent, the congruency effect on reach trajectories emerged for responses to the left or right location but not for responses to the centre location. According to the authors, this observation reflects the impact of stimulus-level conflict which is a delay in response selection and thus, indicating that reach trajectories are not exclusively affected by response conflicts.

Theoretically, the question of whether and how S–S conflict affects dynamically generated responses is informative based on the following argument. If S–S conflict were to emerge in a similar way to how S–R conflicts appear (mainly: via increased curvature toward the incorrect response on incongruent trials), this would imply that information continuously flows from stimulus encoding to response preparation, and eventually into motor action. This would argue against a ‘thresholding’ between stimulus encoding and response preparation, as hypothesised in Kornblum et al.’s (1999) model. It would further require crossing of the ‘cognition-action gap’ highlighted by Calderon et al. (2018) according to which release of a movement is dynamically gated based on the perceived amount of conflict during stimulus encoding and response preparation. Instead, S–S effects emerging in the curvature of dynamic response movements would support the idea that behaviour results from continuous information flow between perceptual, cognitive, and motor processes (Magnuson, 2005).

### The present study

**Fig. 1:**
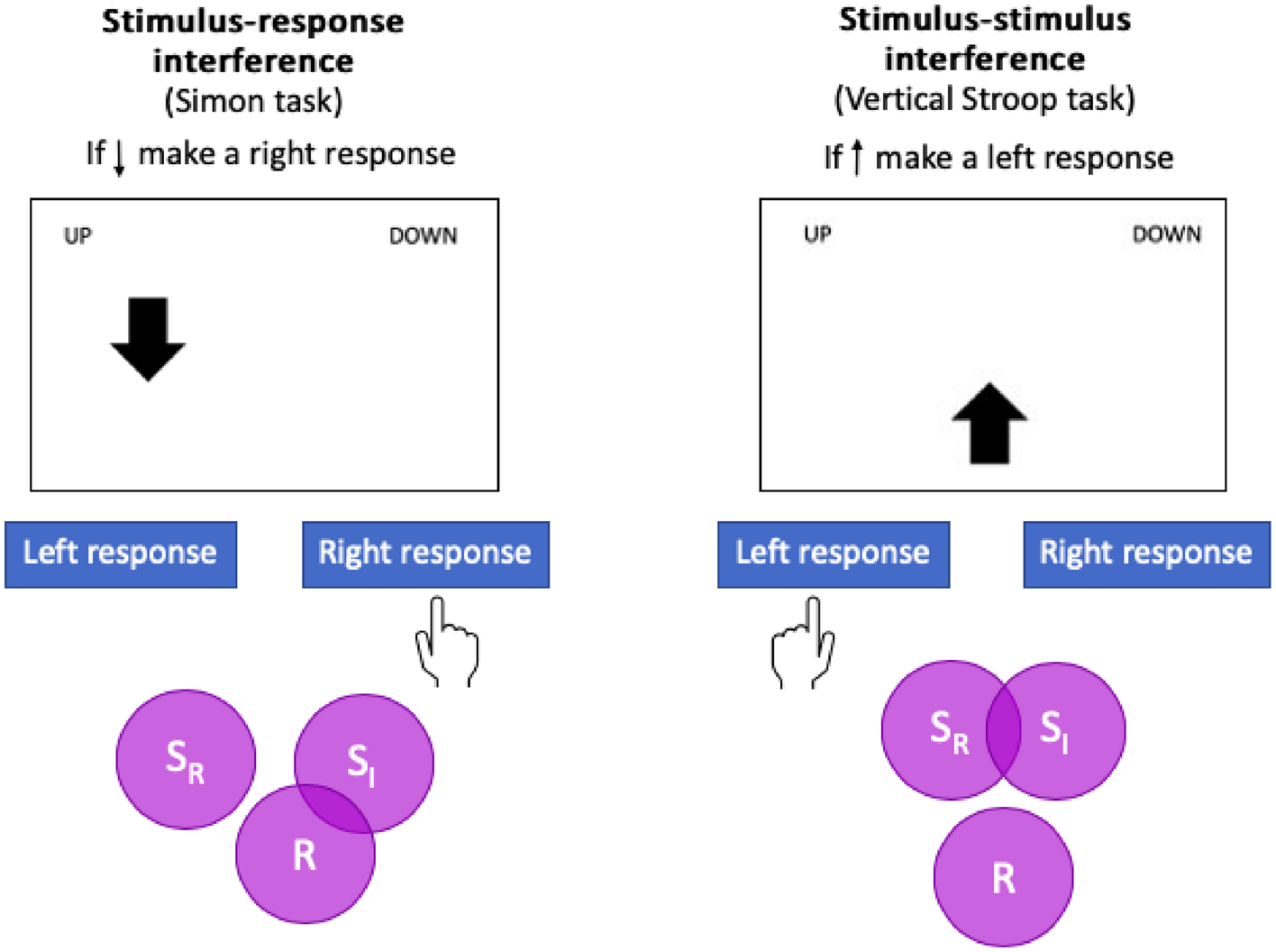
Experimental task. Participants classify up- or downward pointing arrows (in this case, “if ↑ make a left response”). Stimulus–response manipulation: arrows are presented to the left or right of screen centre. Stimulus–stimulus manipulation: arrows are presented above or below the centre. S_R_ = relevant stimulus dimension; S_I_ = irrelevant stimulus dimension; R = response dimension; correct is radiated. Both conflict types show incongruent trials. Adapted from Egner (2008) and Paap et al. (2020)

In the current study, we explored stimulus–stimulus, as well as the more standard stimulus–response based, interference effects and their consequence on the dynamics of mouse tracking. We adopted an integrated task which generates both types of conflict within a single design and the same target stimuli, and with trials from each conflict type randomly intermixed. Figure 1 shows the central manipulation. The task requires participants to map up- or downward pointing arrows onto left-or right responses, with the assignment of direction to response key rotated across participants. The arrows themselves can appear either to the left or right of the centre of the screen, in which case the manipulation generates a stimulus–response (in-)congruency, as in the classic Simon task (a ‘type 3’ conflict in Kornblum’s, 1994, terminology). Alternatively, arrows can appear either above or below the screen centre (Vertical Stroop task) in which case a stimulus–stimulus (in-)congruency is generated (‘type 4’ conflict in Kornblum’s taxonomy).

The spatial arrow task has been used in the literature before. For instance, Li et al. (2014) found S–S and S–R effects of 33 and 37 ms, respectively, with key press responses, and similar effect sizes were reported by Wang et al., (2014; S–S: 30 ms; S–R: 36 ms). By contrast, Paap et al. (2020) reported somewhat larger effects (S–S: 77 ms; S–R: 91 ms) which may have arisen from the fact that a high proportion of congruent to incongruent trials (75/25) were used whereas the earlier studies used even proportions. Inclusion of a high proportion of congruent trials encourages a tonic adaptation of control strategies which results in larger conflict scores than when the proportion is low (e.g., Funes et al., 2010) but it could also induce ‘contingency learning’ in which participants may be able to capitalise on contingencies between stimulus and response properties (e.g., Schmidt et al., 2007). Nevertheless, these results demonstrate that with key press responses, the spatial arrow task generates powerful S–R and S–S effects.^1^

Our first experiment replicated these effects in an online task in which participants responded to the target arrows by pressing one of two response keys on the computer keyboard, operated with the index fingers of both hands. Our second experiment used the same manipulation, but now in an in-person mouse tracking context in which participants initiated each trial by clicking on a “Start” region towards the bottom of the screen, and responded by clicking on one of two “Response” regions orientated in the top left and right corners of the screen. To prevent contingency learning (see above), we included equal proportions of congruent and incongruent trials in both studies. We predicted based on previous mouse tracking studies powerful effects of S–R conflict in errors, response latencies, and (most importantly) in curvature of trajectories toward the incorrect response on incongruent trials. The central question was how potential effects of S–S conflict would emerge in mouse tracking, and how their dynamics would compare to those generated by S–R conflict.

## Experiment 1

### Method

#### Participants

30 participants were recruited online (Female = 23, Male = 7, Mean age = 24.87 years old). Participants were recruited through Prolific and received money for their participation. All participants provided informed consent.

#### Materials, design and procedure

The study was carried out on Gorilla (http://gorilla.sc; Anwyl-Irvine et al., 2020), an online platform that enables researchers to perform psychological experiments online. As described in the Introduction, we used a modified vertical Stroop/Simon task in which participants judged the direction of upward- or downward-pointing arrows, presented either to the left or right of the fixation cross, or up or down. Left- or right-presented arrows formed the S–R (Simon) manipulation, and up- or down-presented arrows formed the S–S (vertical Stroop) manipulation. Trials of both types were randomly intermixed. Participants were instructed to press the “q” and “p” keys on the computer keyboard as responses with the index fingers of their left and right hand. Assignment of up- and down-pointing arrows to response keys was counterbalanced across participants.

Participants completed 32 practice trials, followed by 240 experimental trials, with 120 Simon trials, and 120 vertical Stroop trials. Within each manipulation, 50% of trials were incongruent while 50% were congruent. On each trial, participants saw a fixation cross on a white background for 500 ms in the centre of the screen, followed by a black target arrow. To remind participants the response key assignment, the words “UP” and “DOWN”, formatted in black ink, were located on the top left and top right of the screen. Participants were required to respond within 2000 ms of target onset and if not, the next trial would immediately begin. Participants received instant feedback on whether they made a correct or incorrect response. Arrows were presented as solid black shapes on white background (height: approx. 1° visual angle, width approx. 1.3° visual angle) and were shown to left, right, above or below the fixation dot with their centre displaced by approximately 4° visual angle (all measures are estimates because they depend to some extent on the screen on which participants performed the online experiment). An experimental session took approximately 15 min to complete.

## Results

**Fig. 2:**
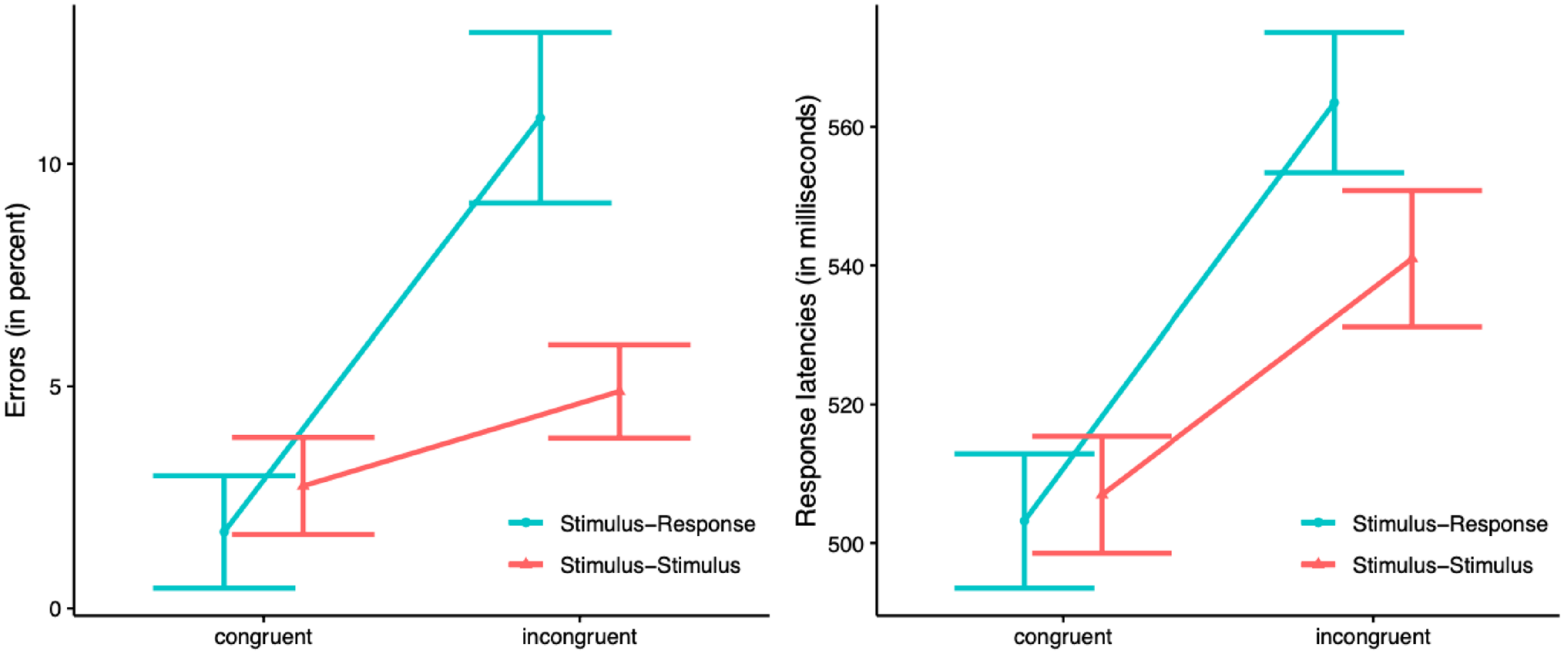
Mean errors rates (in percent; left panel) and response latencies (in milliseconds; right panel) plotted against congruency (stimulus–response vs. stimulus–stimulus conflict type) and congruency (congruent vs. incongruent). Error bars reflect 95% within-participants confidence intervals (Morey, 2008)

Pre-screening revealed that one participant exhibited an error rate larger than 25%, and this participant was removed from the analysis, with 29 participants remaining. Reaction times faster than 150 ms and slower than 1500 ms (1.5%), as well as latencies on error trials (4.4%) were removed from the analysis. Results are shown in Fig. 2.

Errors were analysed via a two-way Analysis of Variance (ANOVA) with the within-participants factors conflict type (S–R vs S–S) and congruency (congruent vs incongruent). Results showed a highly significant main effect of conflict type, *F(*1, 28) = 19.13, *MSE* = 9.92, *p* < 0.001, of congruency, *F*(1, 28) = 54.67, *MSE* = 17.35, *p* < 0.001, and a highly significant interaction between congruency and conflict type, *F*(1, 28) = 31.29, *MSE* = 11.96, *p* < 0.001. The effect of congruency was significant for the S–R conflict type (9.3%), *t*(28) = 7.50, *p* < 0.001; as well as for the S–S conflict type (2.1%), *t*(28) = 3.08, *p* = 0.005. A parallel ANOVA conducted on the response latencies revealed a significant main effect of conflict type, *F*(1, 28) = 9.33, *MSE* = 273, *p* = 0.005 and congruency, *F*(1, 28) = 110.84, *MSE* = 582, *p* < 0.001, and a significant interaction, *F*(1, 28) = 4.86, *MSE* = 1030, *p* = 0.036. The effect of congruency was significant for the S–R type (60 ms), *t(28*) = 7.75, *p* < 0.001, as well as for the S–S type (34 ms), *t*(28) = 4.78, *p* < 0.001.

## Discussion

This study exhibited highly significant S–R and S–S congruency effects both in response latencies and error rates. Regarding latencies, the S–S effect (34 ms) reported in our study compares well with previous studies using a similar arrow task, for example, Li et al., (2014; S–S: 33 ms; S–R: 37 ms) and Wang et al., (2014; S–S: 30 ms; S–R: 36 ms). However, in comparison to these studies, the S–R effect (60 ms) in our study is noticeably larger. The reason for this discrepancy is presently unclear, nonetheless we reliably captured S–S and S–R congruency effects in key press responses in the arrow task. We now tackled the central question of how these effects emerge in mouse tracking. As outlined in the Introduction, numerous mouse tracking studies have used S–R manipulations and observed more curved response trajectories for incongruent than congruent trials. The central question is whether this is also the case for S–S conflicts which are generated via conflict between relevant and irrelevant *stimulus* dimensions but do not involve the response dimension.

## Experiment 2

### Method

#### Participants

28 University of Bristol undergraduate students (Female = 21, Male = 6, Non-Binary = 1, average age = 19.71 years old) were recruited. They were rewarded with experimental credits for their participation and were tested individually in a quiet laboratory setting.

#### Materials, design and procedure

This experiment replicated the online study reported as Experiment 1 as closely as possible in terms of experimental design and procedure. However, *MouseTracker* software, developed by Freeman and Ambady (2010), was used to run the task. Participants were seated approximately 60 cm from a computer screen (23 inch Dell P2319H flat screen monitor with screen resolution 1920 × 1080). Participants initiated a trial by clicking on a grey box (192 × 108 pixels; 5.1 × 2.9 cm) in the bottom centre of the screen. A fixation cross appeared in the centre of the screen for 500 ms. Afterwards, the arrows were presented as solid black shapes on white background (170 × 130 pixels; 4.5 × 3.4 cm) and were shown to left, right, above or below the fixation dot with their centre displaced by approximately 260 pixels (6.9 cm). Participants made a response by clicking on one of two response fields (288 × 144 pixels; 7.6 × 3.8 cm) on either the top left or the top right corner of the screen. Response boxes were labelled with UP or DOWN to indicate the corresponding response. MouseTracker recorded x and y coordinates of the trajectory of the mouse movement every 16 ms for the duration of the trial (2500 ms). Participants were not able to move their mouse before the arrow was displayed. This was to prevent recording negative initiation times which would be difficult to interpret. Additionally, to ensure that we are measuring online processing and hence, participants are not using the strategy of fully committing to a decision before the moving the mouse, we implemented an initiation time deadline of 250 ms. Cursor speed was set in MouseTracker to a value of 12 (with 1 the slowest and 20 the fastest setting).

## Results

Data were processed in *R* (R Core team, 2021) using the package *mousetrap* (Kieslich, Henninger, Wulf et al., 2019). For each trial, initiation time was calculated as the time at which participants began moving the mouse, calculated relative to the onset of the target arrow (in milliseconds). Response latencies were determined as the time between target onset and the time at which participants clicked on one of the response boxes (in milliseconds). Errors were calculated as responses toward the incorrect response region. As a measure of curvature of a movement trajectory, we used “Area under curve” (AUC), the geometric area between the trajectory and a straight line proceeding directly from the start to the response region, calculated in pixels (Kieslich, Wulff, Henninger & Haslbeck, 2019). To avoid very large numbers, these were subsequently divided by 1000.

Trials with response latencies faster than 0 ms and slower than 2000 ms (1.6%) were excluded from the analysis, as were trials with initiation times slower than 500 ms (2.6%). All trajectories were time-normalised into 101 time steps and flipped to appear pointing toward the left response box. Finally, in previous experiments using MouseTracker we observed that participants clicking on the “Start” region often generate unintentional very small cursor movements which can be mistaken for genuine movement initiation times. Hence, in the current study we implemented an initiation threshold such that the first time sample with a cursor movement larger than 36 pixels was taken as the initiation time. This specific value was chosen because upon clicking on “Start”, MouseTracker moves the cursor to the centre of the start region which has a height of 72 pixels. Hence, initiation was conceptualised as the time at which the cursor left a virtual circle around the starting position with a radius of 36 pixels (cf. Ye & Damian, 2023).

**Fig. 3:**
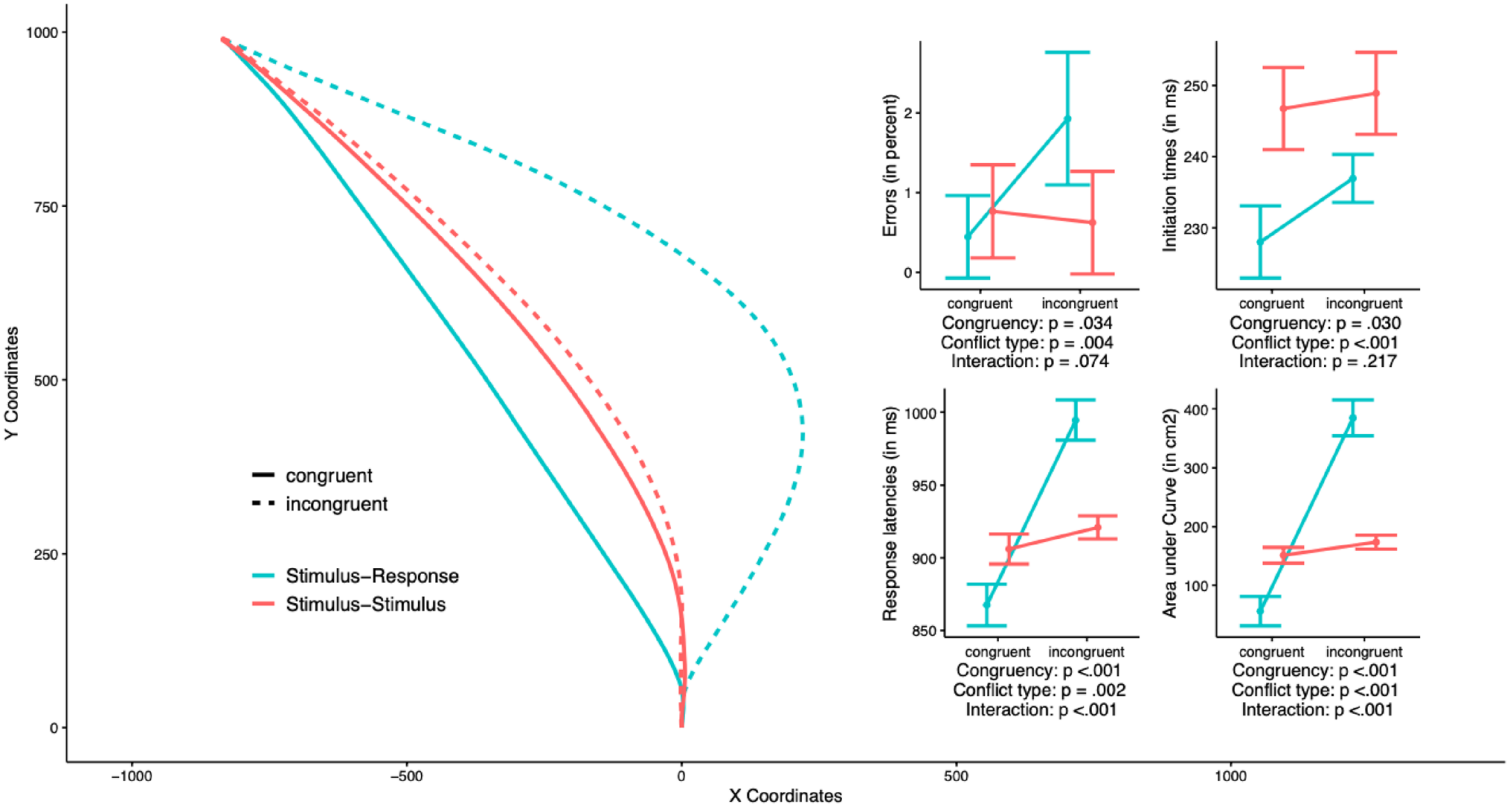
Experiment 2. Left side: average time-normalised trajectories by congruency (congruent vs. incongruent) and type of conflict (Stimulus–Response vs. Stimulus–Stimulus). The right side shows performance in terms of errors, initiation times, response latencies, and curvature (area under curve). Error bars reflect 95% within-participants confidence intervals (Morey, 2008)

Figure 3 shows average mouse movement trajectories on the left side, and inset panels on the right side show the four dependent measures (error rates, initiation times, response latencies, curvature) which were analysed via two-way ANOVAs with conflict type (S–S vs S–R) and congruency (congruent vs incongruent). The inset panels on the right side also show the ANOVA results, with the *p* values corresponding to conflict type, congruency, and the interaction reported below each panel (detailed statistics can be found in Appendix A). Average trajectories are less straight in the incongruent condition, when compared to the congruent condition, and this was the case not only for the S–R conflict type, but also (and critically) for the S–S conflict type.

An interaction between type of conflict and congruency was found in response latencies, and AUC but not in initiation times and errors. Simple effects of congruency carried out for each conflict type separately showed that for the S–R task, the congruency effect was significant in the errors, 1.48%; *t*(27) = 2.64, *p* = 0.0137, response latencies, 127 ms; *t*(27) = 11.86, *p* < 0.001, initiation times, 9 ms, *t*(27) = 3.21, *p* = 0.003, and curvature, *t*(27) = 14.65, *p* < 0.001. For the S–S task, congruency was not significant in the errors,  – 0.14%; *t*(27) = 0.29, *p* = 0.776, and initiation times, 2 ms, t(27) = 0.50, p = 0.621, but it was significant in response latencies, 15 ms; *t*(27) = 2.53, *p* = 0.017, and curvature, *t*(27) = 2.68, *p* = 0.012.

As can be seen in Fig. 3, the average trajectory corresponding to the S–R ‘incongruent’ condition points to the incorrect response in its early stage, then diverts course and finally arrives at the correct response. On a broad level, this ‘change of mind’ (CoM) indicates that the decision had not been completed when movement was initiated but was revised during action execution, and that the incorrect response was previously activated. By contrast, for the S–S ‘incongruent’ condition it is less clear whether CoM took place, and hence whether incongruency results in an attraction towards the incorrect response. To explore this issue, we performed a ‘cluster analysis’ in which each raw trajectory was mapped onto one of five ‘prototypes’, with two of them (‘straight’ and ‘curved) involving no temporary diversion toward the wrong response, and the three remaining ones involving various forms of CoM (see Wulff et al., 2019, for details). An ANOVA conducted on the proportion of CoM trials showed main effects of type of conflict, congruency, and an interaction (all *p*s < 0.001). For the S–R trials we observed a dramatic increase of CoM trials in the incongruent relative to the congruent condition (0.07 vs 0.55, respectively; *t(27)* = 14.23, *p* < 0.001); by comparison, for S–S trials the proportion of CoM trials differed less strongly and the statistical comparison was not significant (0.17 vs 0.20; *t(27)* = 1.87, *p* = 0.072). These results highlight the powerful attraction towards the incorrect response on S–R incongruent trials (which is exactly as expected given that the conflict involves the response dimension); by contrast, the results regarding whether incongruent S–S trials also involve activation of the incorrect response are not particularly clear.

## General discussion

In our experiments, we used an integrated task previously used in the literature (e.g., Li et al., 2014) which allows the generation of stimulus–response and stimulus–stimulus compatibility effects. Experiment 1 used key press responses and confirmed that the integrated task allowed us to reliably capture both S–S and S–R effects, in line with previous studies which had used a similar manipulation (e.g., Li et al., 2014; Paap et al., 2020; Wang et al., 2014). In Experiment 2, responses were made via dynamic computer mouse movements, and the main aim was to compare and contrast the way in which S–R and S–S effects emerge in responses of this type. As highlighted in the Introduction, S–R manipulations have been widely used in conjunction with mouse tracking and typically emerge not only in response latencies, but also in the curvature of the movement trajectories. The critical question was whether S–S manipulations would emerge in a similar fashion to S–R conflicts. The answer was positive: S–S effects also emerged in the curvature of response movements, although in reduced magnitude compared to S–R effects.

A methodological implication of our results is that mouse tracking can clearly be used to investigate S–S conflict. However, our findings are theoretically informative as well. As described in the Introduction, the fact that an S–R conflict consistently affects the characteristics of ‘dynamic’ responses such as mouse tracking or reaching suggests that cognition and action are not ‘staged’, i.e., motor execution begins before a decision has been fully completed. This finding is broadly in line with cognitive theories in which mind, body and environment dynamically interact (e.g., Spivey & Dale, 2004). More specifically, if there is a ‘threshold’ between cognition and action (e.g., Calderon et al., 2015) it is evidently set relatively low in our mouse tracking task such that motor activation is released at a quite early stage of the decision-making process. Our novel finding is that S–S compatibility affects dynamic responses. For an effect such as our current S–S manipulation (which resides at the cognitive stage of stimulus encoding) to emerge in response characteristics, it has to be assumed that stimulus encoding and response preparation are ‘cascaded’ and processing is not staged: a S–S conflict cascades into response preparation, and finally emerges in motor execution. This assumption is at odds with theoretical models such as Kornblum’s (1999) DO model which propose thresholding between stimulus encoding and response preparation. If this was true, the prediction would have been that only S–R effects appear in curvatures. This is because proponents of a model of this type would argue that a S–S conflict is resolved at the stimulus encoding stage before movement initiation while S–R conflict would be resolved during the response preparation stage. By contrast, our findings indicate that both S–S and S–R effects can be observed in mouse trajectories. Therefore, our results are in line with ‘continuous flow’ models which bridge the gap between cognition and action (Erb et al., 2021; Eriksen & Schultz, 1979).

A potential counterpoint that can be made is that curved trajectories do not reflect online corrections in movement. For example, Song and Nakayama (2008) observed no difference in response latencies (note, the authors used the term “total time”) between curved and straight trajectories. As a result, trajectories appeared to be comparable in terms of efficiency and in turn, the authors concluded that corrective movements plans are generated before movement is initiated. This conclusion would be at odds with “continuous flow” models which hold that movement can be executed before a decision has been completed. However, our data in Experiment 2 are not in line with Song and Nakayama’s findings since it appears that curved trajectories are less efficient (indexed by response latencies) than straight trajectories. To demonstrate, on Fig. 3, when comparing the average trajectories of S–R congruent trials with S–R incongruent trials, the former is noticeably straighter than the latter. If curved trajectories reflect corrective movement plans that were made before a participant started moving the mouse, then we should expect to observe similar response latencies between these two trial types. However, as can be seen on the response latencies graph on Fig. 3, average responses latencies are significantly longer for S–R incongruent than S–R congruent trials. Therefore, for our data, curved trajectories or larger AUCs do appear to reflect online corrections and thus imply that participants are moving their mouse before fully committing to a decision.

It is important to embed the results of the current study in a wider literature on decision making and action. Imagine a hypothetical result of our Experiment 2 in which stimulus–stimulus incompatibility resulted in a movement trajectory which compared to the compatible condition, was simply shifted in time (with slower initiation and response times) but generated trajectories of identical shape. As outlined in the Introduction, every situation which requires a decision via action involves some sort of competition between response alternatives. The hypothetical scenario would require that (a) this competition took place, and was resolved, in an abstract decision space rather than between response alternatives, and that (b) action commenced only after the decision was completed. Although such a scenario is not impossible and could in fact be predicted by ‘serial’ models such as those by Kornblum et al. (1999), more recent theorising tends to conceptualise decision making and action as a graded continuum. For instance, Wispinski et al. (2020) provide an extensive review and interpretation of a wealth of behavioural and neural evidence and suggest that the relationship between decision making and action is best characterised as a continuous and graded process which traverses a ‘landscape’ of behavioural options, from presentation until movement has been completed. This relationship can more clearly be captured using dynamic responses as opposed to ballistic ones. Under this theoretical perspective, the central finding of our second experiment which involved a dynamic rather than ballistic response method (mouse tracking), the results regarding the stimulus–stimulus compatibility condition were maybe predictable. Indeed, perhaps in this view, the surprising aspect of our findings is that stimulus–stimulus incompatibility had merely a rather subtle effect on movement trajectories. As highlighted in the Introduction, the bulk of relevant work on mouse movement trajectories has involved variations of stimulus–response compatibilities, and here their consequences for trajectories are well-documented in numerous studies. The results of our Experiment 2 suggest that indeed, the bulk of effects on movement curvatures derives from directly competing response options, with stimulus–stimulus conflicts generating much smaller (but still reliable) consequences for trajectories. Considering the results of the change-of-mind (CoM) analysis, it is unclear whether stimulus–stimulus conflict results in an attraction towards the incorrect response or in a delay in decision making where participants move the mouse forward between the two response options for a longer time. This differs from stimulus–response conflict where it appears to be clear that conflict results in an increase in attraction towards the incorrect response.

Given that our mouse tracking experiment yielded evidence that stimulus–stimulus conflict can affect the characteristics of response movements, future research may investigate whether our findings generalise to other tasks and stimuli. For instance, in a related task used in the neuroscientific literature to disentangle stimulus from response-based conflicts (e.g., Marinkovic et al., 2012), participants classify four colour patches into two responses (e.g., press ‘left’ if green or red; press ‘right’ if blue or yellow). Target colour patches are ‘flanked’ by two distractor patches of the same colour, which can either be congruent (e.g., a red target flanked by red distractors), stimulus incongruent (a red target flanked by green distractors), or response incongruent (a red target flanked by yellow distractors). Response latencies show a gradient, with slower stimulus incongruent than congruent responses, and slower response incongruent than stimulus incongruent responses. The prediction based on our current findings is that in a mouse tracking version of this task, average trajectories associated with the three conditions will follow the same pattern, with trajectories for the response incongruent condition showing a sizeable deflection towards the incorrect response, and trajectories for the stimulus incongruent condition still showing some deviation from the congruent condition (but probably less so than in the stimulus incongruent condition). The same logic applies to the Eriksen task with letter flankers (Eriksen & Eriksen, 1974). Here, participants classified four target letters into two response categories (H and K, or S and C). Flanking distractor letters could be stimulus-incongruent (e.g., KKKKHKKK) or response-incongruent (SSSHSSS), and relative to a condition in which targets and flankers were identical, latencies showed a gradient with somewhat slower latencies for stimulus-incongruent flankers, and substantially slower latencies for response-incongruent flankers. Again, we predict that in a mouse tracking study, the same gradient should appear in the curvature of average response trajectories.

As summarised in the Introduction, Li et al. (2014) factorially crossed the S–R and S–S manipulation with the same spatial arrow task used in the current study, and found strict additivity in the conflict scores. A potential further experiment would be to implement this manipulation in a mouse tracking task; our prediction, based on our findings, is that interactivity rather than additivity would be observed in curvatures. This is because we found both S–S and S–R effects in curvatures, with the inference that stimulus encoding and response preparation are closely related and both cascade into motor execution. Having said that, considering that the magnitude of the S–S effects in response latencies for Experiment 2 (15 ms) was relatively small, it is possible that no interaction would be found in latencies and/or curvatures, because according to Hommel (1997) and Sanders (1980) interactions require relatively large base effects to be reliably observed.

In conclusion, our current study is to our knowledge the first to demonstrate ‘pure’ S–S effects emerging in the curvature of response trajectories in a mouse tracking task. A major theoretical implication is that stimulus encoding and response preparation are closely related (i.e., processing of the former ‘cascades’ into the latter) and both cross the gap between cognition and action (i.e., characteristics of decision making emerge in motor characteristics). Future research should use the mouse tracking paradigm to further investigate cognitive control via the exploration of S–S and S–R manipulations.

## Data Availability

The data used to generate the results mentioned in this study are available from the corresponding author upon request.

## Appendix A

### Statistical results of Experiment 2

**Table:**

	df	MSE	F	η^2^	*p*
*Errors*					
Congruency	1, 27	2.52	5.01	0.156	0.034
Conflict type	1, 27	0.71	9.61	0.263	0.004
Congruency × Conflict type	1, 27	5.34	3.46	0.114	0.074
*Initiation times*					
Congruency	1, 27	163.72	5.24	0.163	0.030
Conflict type	1, 27	152.16	43.38	0.616	< 0.001
Congruency × Conflict type	1, 27	201.19	1.60	0.056	0.217
*Response latencies*					
Congruency	1, 27	939.66	150.23	0.848	< 0.001
Conflict type	1, 27	723.64	11.99	0.308	0.002
Congruency × Conflict type	1, 27	1151.40	76.62	0.739	< 0.001
*Area under curve*					
Congruency	1, 27	4007.86	215.96	0.889	< 0.001
Conflict type	1, 27	1289.27	73.44	0.731	< 0.001
Congruency × Conflict type	1, 27	4041.90	162.93	0.858	< 0.001

## Footnotes

1 See also Meier and Kane (2015) for a further study which manipulated S–S and S–R conflict type by using vertically and horizontally displaced up- and down arrows, and in which the relation between conflict resolution and individuals’ working memory capacity was explored.
